# Enhanced 3-D GM-MAC Protocol for Guaranteeing Stability and Energy Efficiency of IoT Mobile Sensor Networks

**DOI:** 10.3390/s19143230

**Published:** 2019-07-23

**Authors:** Yoonkyung Jang, Ahreum Shin, Intae Ryoo

**Affiliations:** Determent of Computer Science and Engineering, Kyung Hee University, Gyeonggi-do 17104, Korea

**Keywords:** wireless mobile sensor networks, 3-D GM-MAC, Internet of Things, energy efficiency, stability, fixed sensor node, mobile sensor node, buffer threshold, group number reset

## Abstract

In wireless sensor networks, energy efficiency is important because sensor nodes have limited energy. 3-dimensional group management medium access control (3-D GM-MAC) is an attractive MAC protocol for application to the Internet of Things (IoT) environment with various sensors. 3-D GM-MAC outperforms the existing MAC schemes in terms of energy efficiency, but has some stability issues. In this paper, methods that improve the stability and transmission performance of 3-D GM-MAC are proposed. A buffer management scheme for sensor nodes is newly proposed. Fixed sensor nodes that have a higher priority than the mobile sensor nodes in determining the group numbers that were added, and an advanced group number management scheme was introduced. The proposed methods were simulated and analyzed. The newly derived buffer threshold had a similar energy efficiency to the original 3-D GM-MAC, but improved performance in the aspects of data loss rate and data collection rate. Data delay was not included in the comparison factors as 3-D GM-MAC targets non-real-time applications. When using fixed sensor nodes, the number of group number resets is reduced by about 43.4% and energy efficiency increased by about 10%. Advanced group number management improved energy efficiency by about 23.4%. In addition, the advanced group number management with periodical group number resets of the entire sensor nodes showed about a 48.9% improvement in energy efficiency.

## 1. Introduction

As information and communications technologies, as well as computing technologies including various sensors, low-power connections of things, big-data analysis, and artificial intelligence, have been advancing rapidly, the sensor-based Internet of Things (IoT) technology, which is believed to lead the “fourth industrial revolution” is receiving considerable attention. Recently, with the onset of the IoT age, various sensors have been developed; these sensors are located in different environments and collect the necessary information [1]. The information collected through sensors is sent to a gateway or a sink by using a variety of wireless communication technologies; afterward, by going through the analysis and processing processes, useful information is provided to users [2].

Sensor nodes that constitute wireless sensor networks (WSNs) [3,4] can be placed all over a house and automate operations of various devices such as TVs, lights, and air conditioners [5], or can be directly attached to the human body and monitor the health state of the wearer [6]. In addition, sensors can be attached to a variety of equipment in a factory and monitor and control the status of the factory in real time and the machines that manufacture products [7]. Furthermore, they are used to rapidly detect earthquakes that result in serious disasters and send warnings for danger [1], and are placed in the ocean and used for ocean research or military purposes [8].

To use sensor-based IoT technology appropriately, there are several challenges that must be solved. First, most sensors operate with limited energy resources (batteries), and for the replacement of sensor batteries, there are many constraining factors and an enormous cost is incurred [9]. It is difficult for a person to change the batteries in an area that is difficult to access. Furthermore, disposing of waste batteries that have been drained of energy is a significant environmental issue. Second, if the battery life of an arbitrary sensor ends, it may affect the operation of the whole WSN system. This problem is even more difficult to resolve when the sensor has mobility. Third, as the majority of wireless communication technologies used for data transmission between sensors were not originally developed for the purpose of a sensor-based IoT environment, it is less desirable to apply them to a sensor-based IoT environment from the points of view of energy efficiency and sensor network stability. In the end, when wireless sensors operating with limited energy sources send sensed data to a sink, energy-efficient wireless medium access control technology should be used, because it can maximize not only the operating life of each sensor, but also the operation life of the WSN to which the corresponding sensors belong.

To date, many studies have been conducted to improve the energy efficiency of wireless MAC protocols. First, in initial versions, MAC protocols targeted an environment in which sensor nodes and a sink are fixed such as sensor-MAC (S-MAC) [10], timeout-MAC (T-MAC) [11], and pattern-MAC (P-MAC) [12]. By controlling the operating cycle of the sensor relatively simply, these protocols can achieve energy efficiency for each sensor itself, but the operating life of the WSN is not taken into consideration. Furthermore, they are limited in terms of their application to the recent IoT environment, where there is an increasing demand for sensor nodes with mobility. However, recently, studies have been conducted for mobile sensor nodes and fixed sinks [13,14]. In these studies, the application to mobile sensor nodes is easy, but the life of the sensor nodes around a sink is noticeably reduced, thereby affecting the life of the WSN itself.

To overcome these problems, studies have been actively carried out for mobile sensor nodes and mobile sinks. To improve the quality of service, one study changed the data transmission cycle by using the mobility of mobile sinks [15]. In addition, a study was conducted for sensor nodes saving data temporarily based on the location of the mobile sink. Mobile sensor nodes can solve the energy consumption imbalance problem between sensor nodes [16]. Studies have also been conducted for multiple sinks to collect data efficiently in a wider area [17,18]. Although studies for mobile sinks have improved energy efficiency, they have many drawbacks with respect to their application to sensor devices targeting a 3D environment.

As spaces using sensors are becoming more diverse, such as underwater and in the Earth’s atmosphere, the height as well as the length and width have become factors that cannot be ignored. Height is important for MAC protocols targeting a 2D environment (x, y), and which are inappropriate for applications in which nodes are dispersed in a 3D environment. For example, in an underwater sensor network, the sensor nodes are positioned at different depths from each other depending on the depth of the ocean. For climate monitoring, sensors should also be placed in the atmosphere. Therefore, the need for a MAC protocol that targets 3D environments has emerged [19,20,21].

The 3-dimensional group-management MAC (3-D GM-MAC) [22,23] protocol is designed for circumstances where the sensor nodes and sinks all have mobility. The sensor nodes are assigned with group numbers based on the distance to the sink, and they have a hierarchical structure with a tree shape. A sensor node sends data to the sensor node of the next upper group. The sensor node of the next upper group has a group number that is smaller by one than its group number.

Moreover, 3-D GM-MAC is a simple MAC protocol that can be applied to any application. It can be applied to both a wide space and a narrow space. Furthermore, the task of setting the group number is simple and does not use a complex algorithm. In fact, it can be implemented by slightly modifying the header of an existing packet structure.

Furthermore, with 3-D GM-MAC, all sensor nodes show an identical energy consumption rate. If any of the sensor nodes in the whole sensor network depletes all the energy, it affects the whole network. Therefore, 3-D GM-MAC is a very good MAC protocol from the view of energy efficiency.

However, the current 3-D GM-MAC is a MAC protocol that was created in the early years and has very low stability. In this work, methods were proposed to improve the stability of the 3-D GM-MAC. An equation for setting the buffer threshold of each node was newly derived. The original equation of setting the buffer threshold was based on the group number and volume of a sphere, and the original equation has the problem that, as the group number increases, the buffer threshold value decreases sharply. Therefore, the possibility of a bottleneck phenomenon occurring is large as the data are gathered with nodes that have small group numbers. Therefore, the stability is increased by setting the buffer threshold of each node through an equation that sets a rational buffer threshold value based on a mathematical theory.

Another method of improving the stability of the 3-D GM-MAC is to assign fixed nodes to whole WSNs. Fixed sensor nodes are sensor nodes that do not move. Fixed nodes have a higher reliability than mobile nodes, because the fixed sensor nodes do not move, and, consequently, the possibility of having an incorrect group number is low. When a certain node sets a group number, the stability increases because a node with no mobility receives the group number information and sets it ahead of the nodes that have mobility.

The third method of improving the stability of 3-D GM-MAC is to adopt an advanced method for the management of the group number. When a certain sensor node recognizes a situation in which it cannot perform a communication, it goes through a resetting process to be assigned with a new group number. In most cases, this occurs when a sensor node has moved a long distance and deviated greatly from the existing group number area. When resetting a group number, the original 3-D GM-MAC uses an equation that has a weight on the group number and moving distance. However, such a method increases the possibility of setting an incorrect group number. To reset the group number, the advanced method of resetting the group number adds 1 to the smallest value in the information received from other sensor nodes in the vicinity and uses it. To increase the reliability of this method, the stability is increased by using a method of resetting group numbers periodically for all nodes of the entire network.

This work is organized as follows. In Section 2, the original 3-D GM-MAC and problems stemming from it are described. In Section 3, the method proposed here to increase the stability of 3-D GM-MAC is discussed. Section 4 presents the simulation analysis results. Finally, the conclusions and future study plan are provided in Section 5.

## 2. 3-Dimensional Group Management MAC

There is almost no MAC protocol that considers 3-D in the real world. In the past, the number of devices using the Internet was small. However, as the number of things connected to the Internet has increased exponentially and devices that have large mobility, such as drones, have emerged, the need for a 3D MAC protocol has become an issue. The 3-D GM-MAC protocol [22,23] is suitable for real-world applications. Moreover, 3-D GM-MAC can be applied to situations where sinks, as well as sensor nodes, have mobility. Furthermore, sensor nodes that are mobile at high altitudes are also considered.

An example of a protocol using a cluster structure by grouping sensor nodes, is LEACH. LEACH chooses a cluster head for each cluster. The cluster head collects data from other sensor nodes in the cluster and sends them to the sink. LEACH and 3-D GM-MAC have a similarity in that they use a group structure. However, LEACH must select a cluster head by using a stochastic algorithm in every stage. Furthermore, as regular sensor nodes cannot communicate directly with the sink and only the cluster head communicates with the sink, the corresponding cluster head must be in the communicable range from the sink, and, consequently, there is a limitation to applying LEACH to a wide range of applications [24]. In contrast, 3-D GM-MAC is very simple because it does not use a complex algorithm and can set a group number by only receiving a packet that has the group number information. Furthermore, as data existing far away from the sink are delivered to a gateway that performs the role of a sink through relays of sensor nodes in the next upper group, a wide range of data can also be sufficiently collected by using a small number of gateways.

Another important point of 3-D GM-MAC is shown in the energy consumption of the sensor nodes. The sensor nodes communicating directly with a sink cannot avoid consuming energy rapidly. The 3-D GM-MAC protocol has mobile sensor nodes and sinks. Therefore, the group numbers of the sensor nodes keep changing. The sensor node with the group number 1, which communicates with the sink directly, is continuously changing. The 3-D GM-MAC protocol can equalize the energy consumption of all the sensor nodes.

**Figure 1:**
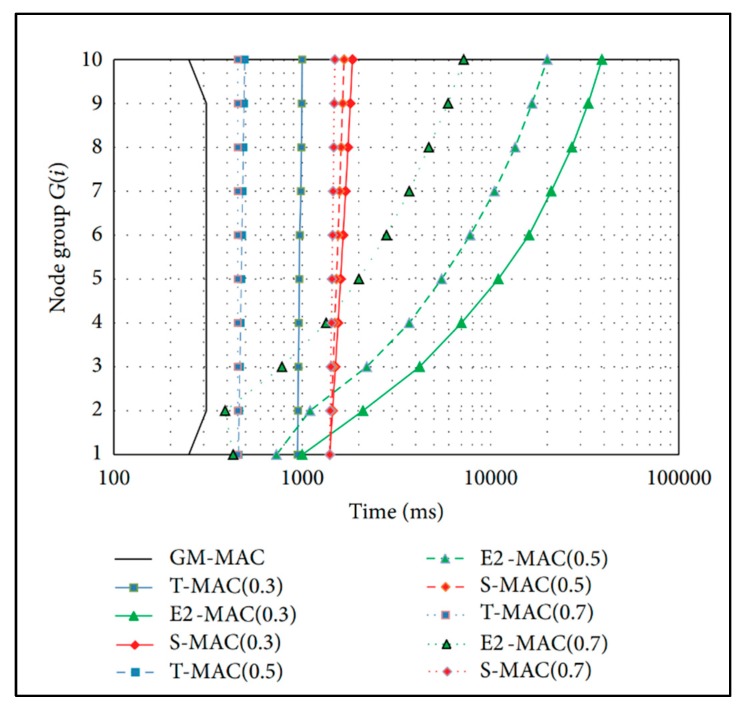
Average active time of sensor nodes.

Figure 1 [25] shows a graph for the comparison of the active times of various MAC protocols. The average active time of sensor nodes in GM-MAC is smallest in comparison with the other MAC protocols. It has been proven that GM-MAC is superior to other MAC protocols and is worth using.

### 2.1. Initial Setting of Group Number and Data Transmission Path

Every sensor node has a group number and a unique ID. The initial group number setting is the first stage of the 3-D GM-MAC. This is performed for every sensor node.

**Figure 2:**
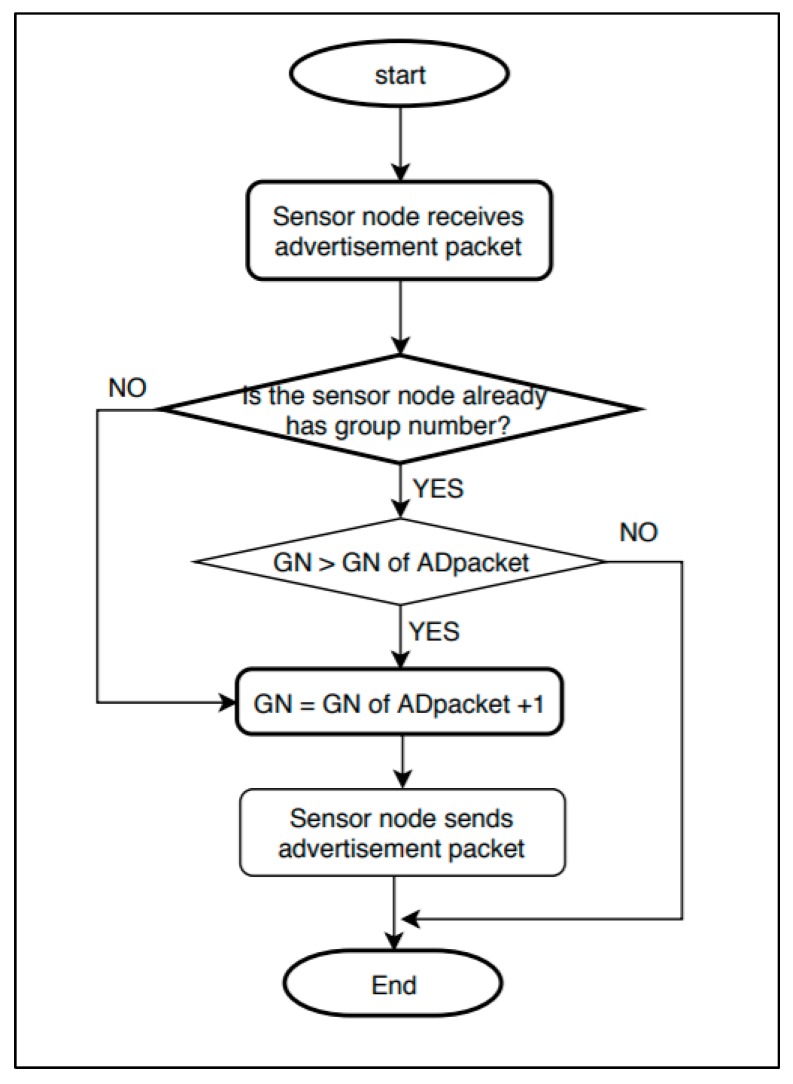
Initial group number setting flowchart.

Figure 2 shows the flowchart for the initial group number setting. “GN” in the figure indicates a group number. “ADpacket” refers to an advertisement packet.

The initial group number setting begins with an advertisement packet from a sink containing the group number information. The initial group number setting is performed through the following procedure.

1. Advertisement packet of the sink: The sink sets its group number as 0. After creating an advertisement packet containing the group number information, it sends the advertisement packet to all sensor nodes within the distance that can be transmitted from it.

2. Sensor node that does not have a group number yet: If a sensor node that has not set the group number receives an advertisement packet, the sensor node uses that advertisement packet. By adding 1 to the received group number, it sets its own group number. Next, it creates and sends an advertisement packet to the surrounding sensor nodes that are within a transmittable distance.

3. Sensor node that already has a group number: If a sensor node that already has a group number receives an advertisement packet, it compares its own group number with the group number of the advertisement packet. If the value of the advertisement packet is smaller, the sensor node updates its own group number by using the group number information of the advertisement packet. Next, it creates and sends an advertisement packet to the surrounding sensor nodes that are within a transmittable distance. In the opposite case, the advertisement packet is ignored.

4. Until the group IDs are set up for all of the sensor nodes, the processes of Steps 2–3 are repeated.

**Figure 3:**
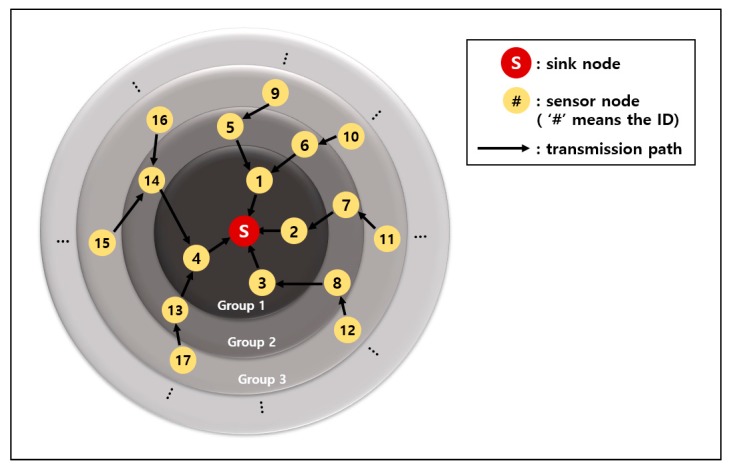
Initial setting of group number and data transmission path.

Figure 3 is an example of a network after the initial group number setting has finished. The red circle with a letter “S” is the sink. The yellow circles with numbers are the sensor nodes. The number is the ID of the sensor node. The black arrow indicates the direction of the data transmission. All data are ultimately gathered at the sink.

### 2.2. Group Number Resetting

If a sensor node becomes located outside the range of the next upper-level sensor that it is communicating with, i.e., the parent node, it can no longer send data. In this situation, the corresponding sensor node resets the group number to resume the data communication. It can be determined whether a certain sensor node is outside the communication range of the parent node if the clear to send (CTS) is not received despite the sensor node sending the request to send (RTS) three times to transmit data to a sensor node of the upper group.

For resetting the group number, a Hello packet and a Reply packet are used. A sensor node that wants to reset the group number sends a Hello packet to other sensor nodes within its communication range. The surrounding sensor nodes that have received the Hello packet send Reply packets containing their group number information. The sensor node resets the group number through the following equations based on the Reply packets.

(1) $$\begin{array}{c} getGroupNum\left(list_{reply}\right)\\ =\{\begin{array}{cc} 1 & ,\mathrm{if}\;\mathrm{receives}\;\mathrm{Reply}\;\mathrm{Packet}\;\mathrm{from}\;\mathrm{Sink}\\ GroupNum\left(list_{reply}\right) & ,\mathrm{if}\;\min(Gn)<GroupNum\left(list_{reply}\right)\\ \min(Gn)+1 & ,\mathrm{if}\;\min(Gn)\geq GroupNum\left(list_{reply}\right) \end{array} \end{array}$$

(2) $$\begin{array}{c} GroupNum\left(list_{reply}\right)\\ =round\left(\frac{\sum^{\text{​}}\left(Gn*\left(1-\frac{\mathrm{movingDistance}}{\mathrm{maxDistance}}\right)*groupWeight\left(Gn\right)\right)}{\sum^{\text{​}}\left(\left(1-\frac{\mathrm{movingDistance}}{\mathrm{maxDistance}}\right)*groupWeight\left(Gn\right)\right)}\right) \end{array}$$

(3) $$groupWeight\left(Gn\right)=\;\frac{1}{3*\left(Gn-1\right)*Gn+1},if\;the\;IoT\;system\;is\;3-D$$

Equation (1) assigns a new group number. If the sensor node that has sent a Hello packet receives a Reply packet from the sink, it sets the group number as 1. When a Reply packet is received from the sink, it means that the sensor node that wants to reset the group number is in the vicinity of the sink.

If the sensor does not receive a Reply packet from the sink, it compares the value obtained from Equation (2) with the smallest value among the values of the Reply packets. If the smallest value among the values of the Reply packets is greater than or equal to the value obtained from Equation (2), the group number is set up by adding 1 to the smallest value among the values of the Reply packets. 

Equation (2) uses an average value that has a weight in the sensor node’s moving distance and group number. The average value is rounded off. It is highly likely that a sensor node with a large moving distance has left the group to which it belonged. Therefore, a small weight is given to this sensor node.

Equation (3) is used for calculating the weight of the group number. The number of sensor nodes in the group number area is proportional to the group range. Therefore, Equation (3) uses the inverse proportion of spherical volume according to the number of groups.

**Figure 4:**
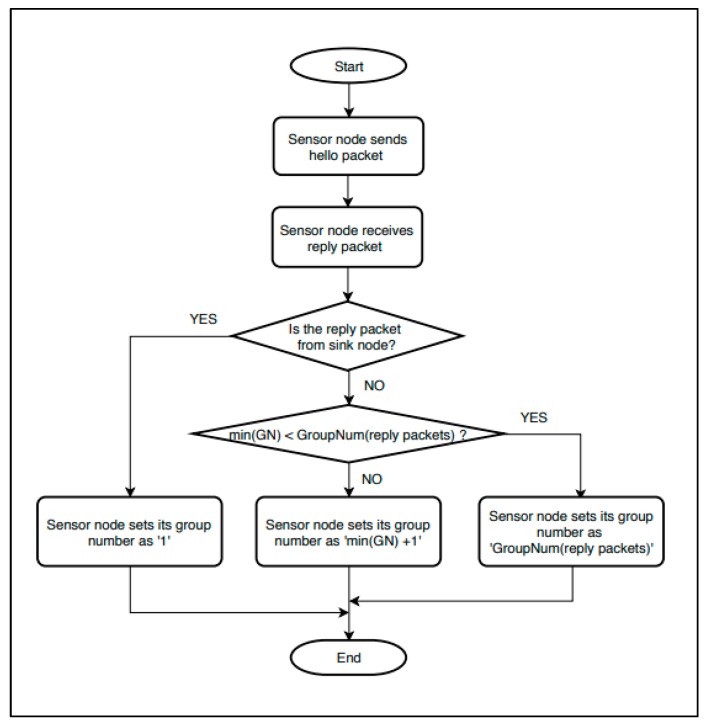
Group number resetting.

Figure 4 is the flowchart for the group number resetting. “GN” refers to a group number. “Reply packets” indicates all combined Reply packets received from the sensor nodes in the communication range.

### 2.3. Buffer Threshold Setting

A buffer threshold is set up based on the group number. A sensor node that has a smaller group number than other sensor nodes with large group numbers needs a large buffer threshold value. The sensor nodes around the sink receive large data from the other sensor nodes. Therefore, the buffers of the sensor nodes around the sink are filled quickly. This requires frequent data transmissions. Each sensor node has an associated buffer threshold, which can be determined using Equation (4) below.

(4) $$B_{i}=\;\beta \;\times \;\frac{B_{t}}{3\times \left(i-1\right)\times i+1}$$

- β: Portion of the total buffer size to be used, 0 ≤ β ≤1;
- B_t_: Total buffer size of sensor node;
- i: Group number of sensor node;
- B_i_: Buffer threshold of sensor node with group number.

The buffer threshold of each sensor node is set up as inversely proportional to the distance to the sink. The sensor node sends data if the data gathered in the buffer exceed the buffer threshold value [22].

**Figure 5:**
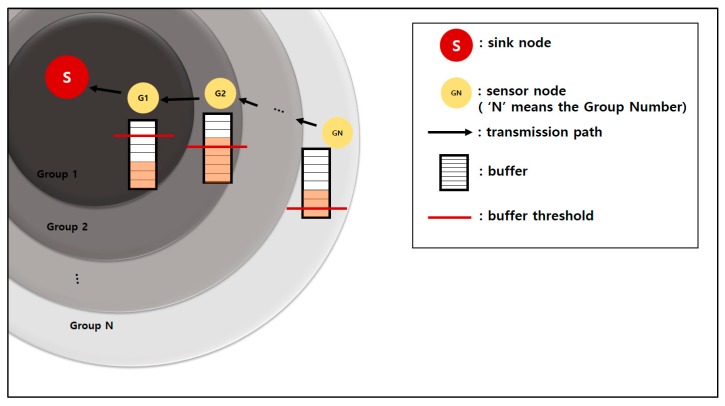
Buffer thresholds for node groups.

Figure 5 shows an example of the buffer thresholds based on the group numbers. The red lines in the figure are the buffer thresholds. The peach color in buffer is collected data. The G1 node has a larger buffer threshold than the G2 node because the sink is closer to the G1 node than the G2 node. 

The G2 node and GN node must send data to the sensor node of the next upper level because the data in their buffers exceed the buffer thresholds. The G1 node must wait until additional data come in and the buffer threshold is exceeded.

## 3. Stability Improvement Proposals for GM-MAC Protocol

The existing 3-D GM-MAC is an early MAC protocol and has a very low stability. Considerably large data loss occurs, and, in some cases, data transmission is impossible because of an incorrect group number setting. To solve these problems, three methods for the stability improvement of the 3-D GM-MAC are proposed. The first method derives a buffer threshold equation to eliminate data loss. The second method uses fixed nodes to increase the reliability of the group number. The last method is an advanced management method of the group number.

### 3.1. Deriving Buffer Threshold Equation

If the original buffer threshold equation from 3-D GM-MAC is used, data loss occurs. Data loss is a significant problem because data integrity cannot be guaranteed. Furthermore, as the group number increases, the buffer threshold becomes extremely small. Consequently, the bottleneck phenomenon occurs easily.

The 3-D GM-MAC protocol is aimed at the reliability of transmission without data loss. For real-time transmission, it is possible to apply 3-D GM-MAC to applications by lowering the buffer threshold, but, in this case, the energy consumption increases greatly. The goal of deriving a new buffer threshold equation is to find an appropriate equation that can guarantee the reliability of data transmission, while solving the bottleneck of the original buffer threshold.

Various experiments have been conducted to derive a better buffer threshold equation.

To conduct the experiments in the refined situation, many conditions are restricted compared to the original 3-D GM-MAC. Table 1 shows the experimental environment for deriving the buffer threshold equation. Every node has no mobility and is placed in the 2-D space.

The 2-D GM-MAC is suitable for WSNs composed of numerous sensor nodes and one gateway. Smart factories and phone line facilities are some examples.

**Figure 6:**
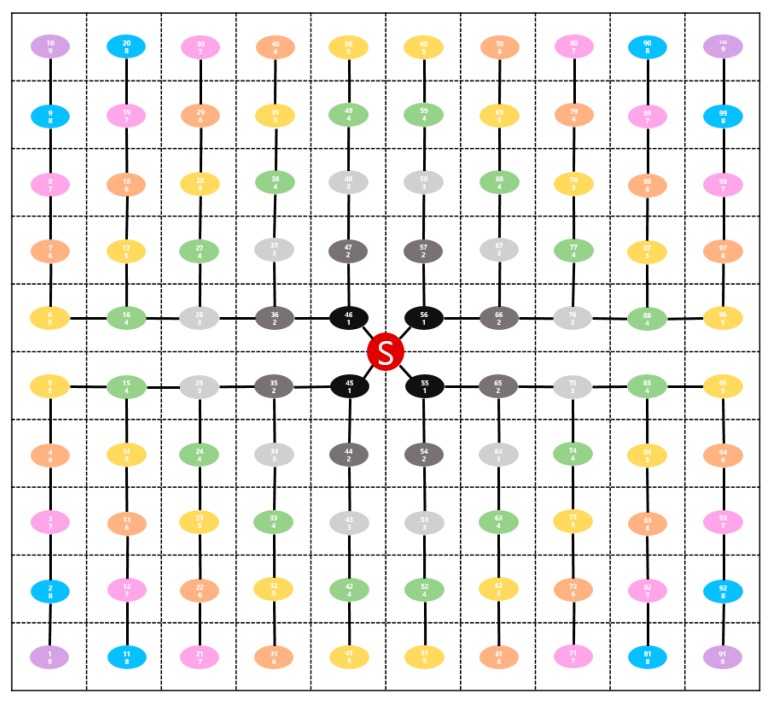
Sample topology for experiments of deriving buffer threshold equation.

Figure 6 shows the topology for deriving the buffer threshold equation. The size of the space is a square with a length of 800 m. The sink is located at the center. The sensor nodes are arranged evenly with regular intervals between them. In each sensor node, the ID and group number are presented. The upper one is the ID, and the lower one is the group number.

(5) $$B_{X}=\;\beta \;\times \;\frac{B_{t}}{aX^{2}+bX+c}$$

(6) $$B_{X}=\;\beta \;\times \;\frac{B_{t}}{\left(a\;*\;log_{b}\left(X+1\right)\right)\;+1\;}$$

- B_X_: Buffer threshold of sensor node with group number X;
- β: Buffer use rate (expectation value), 0 ≤ β ≤ 1;
- B_t_: Total buffer size of sensor node;
- X: Group number of sensor node.

The goal of deriving a new buffer threshold equation is to guarantee energy efficiency while experiencing no data loss. The experiments were conducted for two equations. Equation (5) is for experiments using the polynomial function. Equation (6) is for experiments using the logarithmic function. The goal of the experiments was to find the most suitable values for a, b, and c of each function. For each experiment, 100 random tests were conducted for one value set. The graph below shows the result of the experiments by averaging 100 tests for each experiment. The buffer usage rate was 0.6.

**Figure 7:**
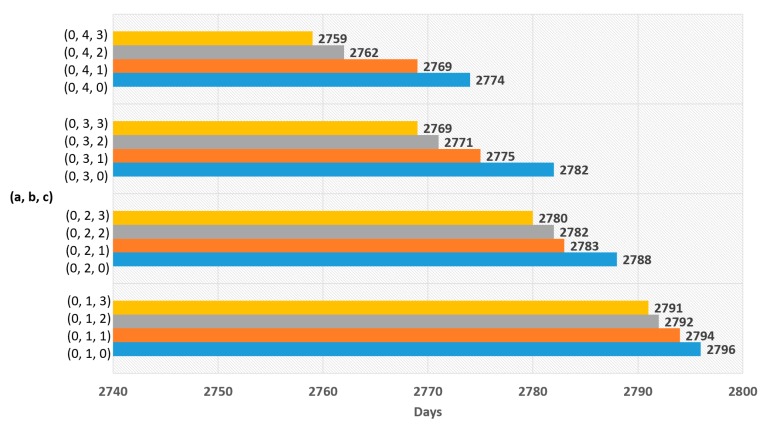
Network lifetime in case of using polynomial function with ‘a’ = 0.

**Figure 8:**
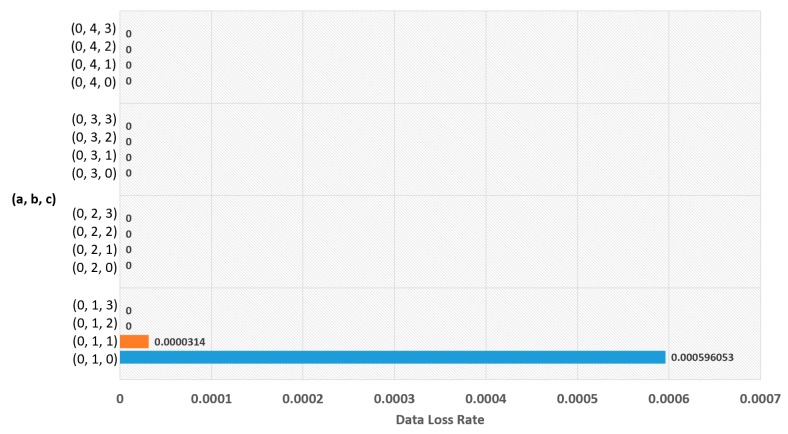
Data loss rate in case of using polynomial function with ‘a’ = 0.

**Figure 9:**
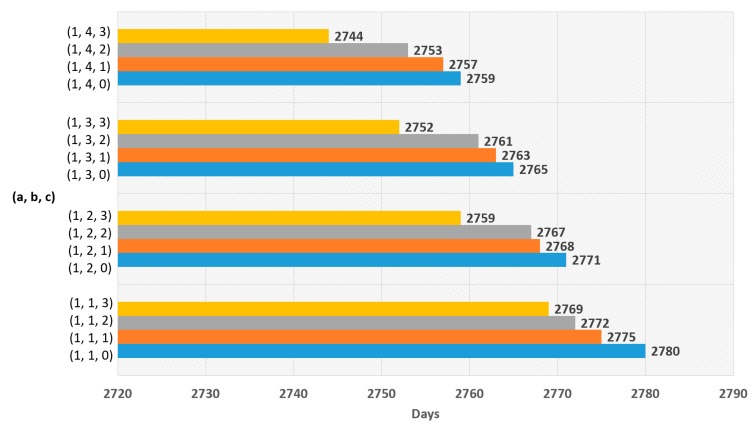
Network lifetime in case of using polynomial function with ‘a’ = 1.

**Figure 10:**
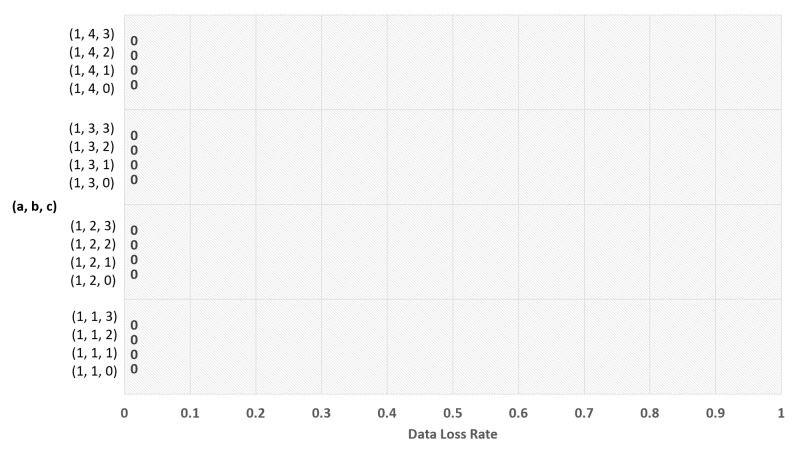
Data loss rate in case of using polynomial function with ‘a’ = 1.

**Figure 11:**
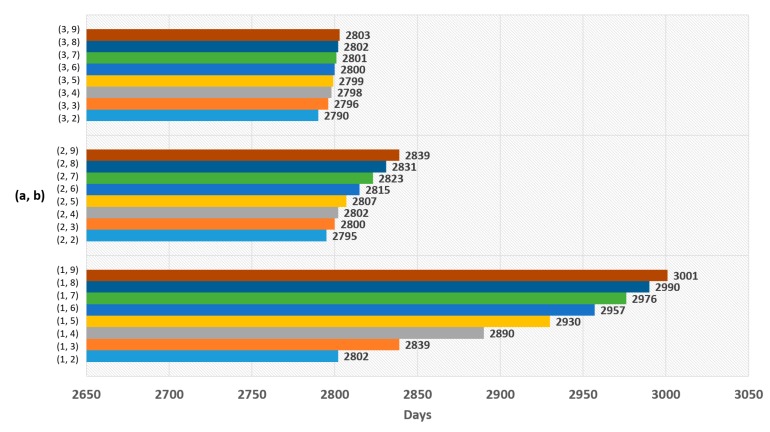
Network lifetime in case of using logarithmic function.

**Figure 12:**
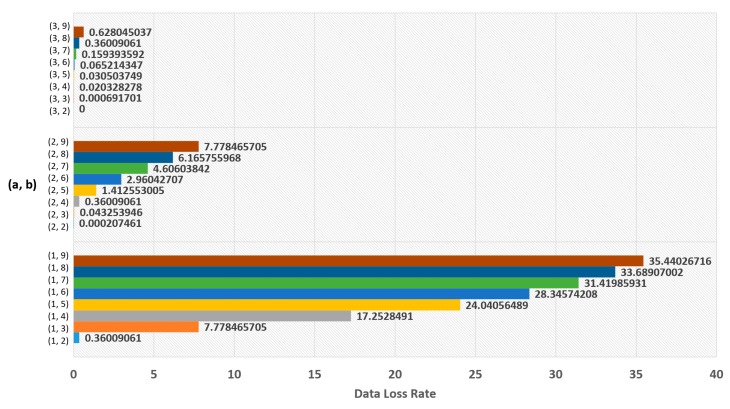
Data loss rate in case of using logarithmic function.

The experimental results for Equation (5) are shown in Figure 7, Figure 8, Figure 9 and Figure 10, and the experimental results for Equation (6) are shown in Figure 11 and Figure 12. The results of each experiment are described with two graphs; one shows the occurrence time point of the sensor node that has completely used up the energy, and the other provides the data loss rate.

When variable a is 0 in Equation (5), Figure 7 shows the occurrence time point when a node has completely run out of energy and Figure 8 shows the data loss rate. The largest value in Figure 7 is 2796 days, which is the result when a = 0, b = 1, and c = 0. However, this value is insignificant because Figure 8 shows that the data were lost with the values. The significant value was the value when a = 0, b = 1, and c = 2, which has no data loss and makes 2792 days.

When the variable a is 1 in Equation (5), Figure 9 shows the time point when a node has completely run out of energy, and Figure 10 shows the data loss rate. The best result in Figure 9 is 2780 days, which is the value when a = 1, b = 1, and c = 0. As there is no data loss, it is a significant result.

For Equation (6), Figure 11 shows the time point when the first node has completely run out of energy, and Figure 12 shows the data loss rate. The best result in Figure 11 is 3001 days, which is the value when a = 1 and b = 9. However, it is an insignificant result because data loss occurred. The significant result was 2790 days, the value when a = 3 and b = 2, which had no data loss.

Summarizing all of the results of the experiments, it was determined that the most suitable buffer threshold was derived by Equation (6) with a = 3 and b = 2. This is because this value showed the best energy efficiency without any data loss. Therefore, Equation (7) is the most suitable buffer threshold equation.

(7) $$B_{X}=\;0.6\;\times \;\frac{B_{t}}{\left(3\;\times \;log_{2}\left(X+1\right)\right)\;+1\;}$$

- B_X_: Buffer threshold of sensor node with group number X;
- B_t_: Total buffer size of the sensor node;
- X: Group number of the sensor node.

If sensor node has mobility, it might cause data loss, because sensor nodes can be concentrated on a certain area. If sensor node has too many child sensor nodes, it will be burdened than when it is in the sample topology in Figure 6. Nevertheless, the data loss rate of the new buffer threshold is much lower than the one of the existing buffer threshold. The new buffer threshold is derived by mathematical experiments, whereas the existing buffer threshold is decided by intuitive guessing.

Also, the new buffer threshold is still worthy, because it can be specified without additional computation by using Equation (7). This is because the buffer threshold is obtained by substituting only the group number into the variable X in Equation (7).

### 3.2. Initial Group Number Setting with Fixed Sensor Node

The 3-D GM-MAC protocol is a MAC protocol that is created by targeting the environment of all sensor nodes and using a sink that has mobility. However, in the real world, although it is true that the number of wireless sensor nodes with mobility is increasing, not every sensor node is moving. There are fixed sensor nodes without mobility. Fixed nodes have high reliability compared with mobile nodes. If fixed nodes are used, the stability of the entire WSNs can be increased.

When group numbers are set up in the original 3-D GM-MAC, the group numbers are set beginning with the sink. The sink also has mobility. While advertisement packets and group number information are being sent and received, all of these tasks are performed between moving nodes. It is impossible to know when a sensor node has set the group number. Therefore, the reliability of the group number information cannot be guaranteed. For example, when a sensor node assigned with the group number 3 communicates information for group number 3 to other nodes after moving to the area of group number 5, whether this information can be trusted or not cannot be determined.

In 3-D GM-MAC using fixed nodes (a method proposed in this work), the group number setting task is divided into the primary setting and secondary setting. In the primary setting, the information of a fixed node has a higher priority than that of a mobile node when setting the group number. The primary setting begins with an advertisement packet of the sink. In the primary setting, mobile nodes do not participate. The advertisement packet of the sink is only sent to the fixed nodes.

A fixed node that receives the group number information from the sink sets its own group number as “1”. Afterward, it produces an advertisement packet containing its own group number information. This advertisement packet is delivered to other fixed nodes existing in the communication range.

If a node that has already set up the group number receives an advertisement packet from another node, it compares the received information with its own group number. If it is less than or equal to its own group number, the advertisement packet is ignored. If not, the node updates its group number by using the advertisement packet. Then, it sends its own advertisement packet to other fixed nodes existing in the communication range. If there is a fixed node that is not assigned a group number in the primary setting, it does not take any action during the primary setting period. This node is assigned a group number in the secondary setting.

The secondary setting begins with an advertisement packet of the sink. If a sensor node with mobility receives the advertisement packet from the sink, it sets its own group number as 1. After the delivery of the sink’s advertisement packet, the fixed nodes that have completed the group number setting send the advertisement packets. If a mobile node that has not yet set up the group number receives the advertisement packet, it sets its own group number by adding 1 to the corresponding information. If a mobile node that already has a group number receives the advertisement packet, it compares its own group number with the advertisement packet information. Unless the information is smaller than its own group number, the advertisement packet is ignored.

The mobile nodes that have updated the group number create their own advertisement packets. These advertisement packets are sent to other sensor nodes existing in the communication range. The fixed nodes with no group number have group numbers in the secondary setting. The WSN system repeats the above process until every sensor node has a group number.

**Figure 13:**
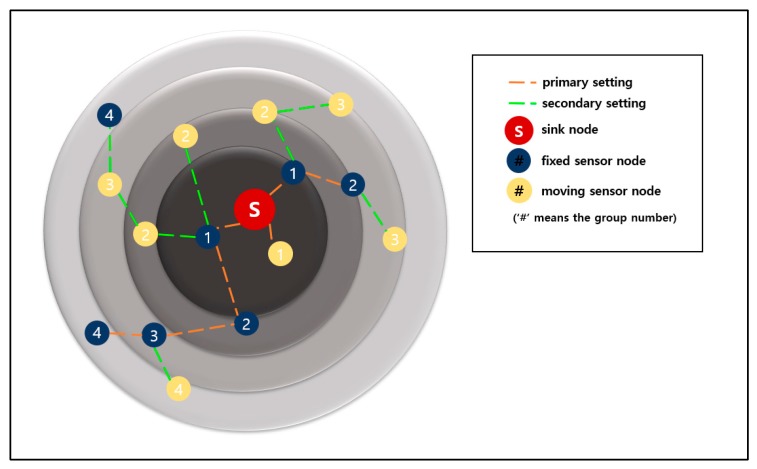
Primary and secondary settings of node group numbers.

Figure 13 shows the primary setting and the secondary setting. The red circle is the sink. The dark blue circles are the fixed sensor nodes, and the yellow circles are the mobile sensor nodes. The orange dotted lines indicate the primary setting, and the green dotted lines indicate the secondary setting.

If the initial group number setting starts, the sink and the fixed nodes send advertisement packets in sequence following the orange dotted lines. Afterward, the mobile sensor nodes participate in this task after the secondary setting has begun. In the secondary setting, the nodes that have updated the group number send the advertisement packets following the green dotted lines.

**Figure 14:**
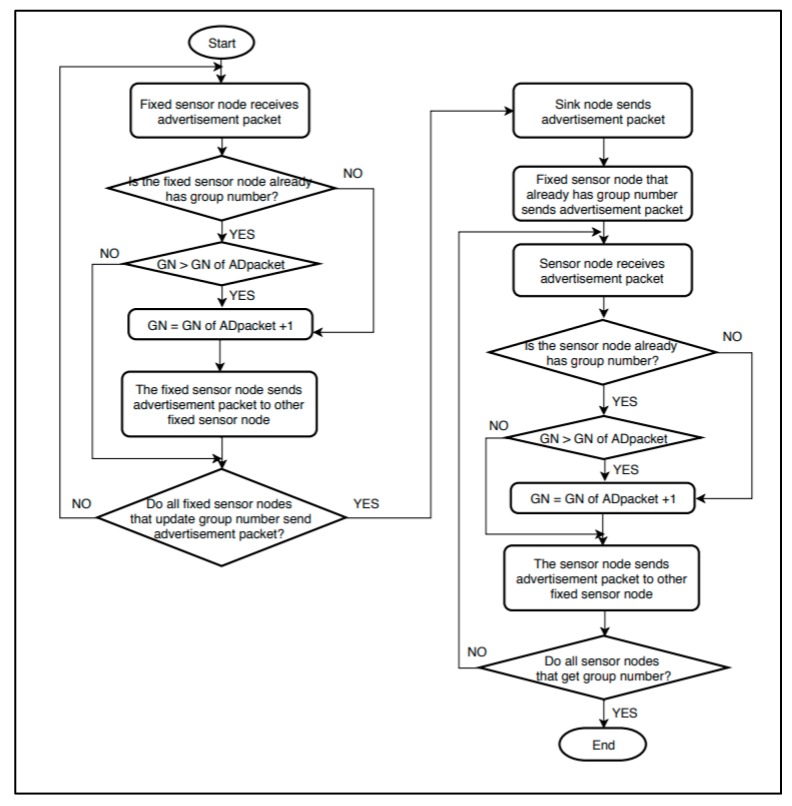
Initial group number setting using fixed sensor nodes.

Figure 14 shows the flowchart for the initial group number setting using fixed sensor nodes. “GN” is a group number and “ADpacket” refers to an advertisement packet. The left side of Figure 14 shows the primary setting process, and the right side shows the secondary setting process.

**Figure 15:**
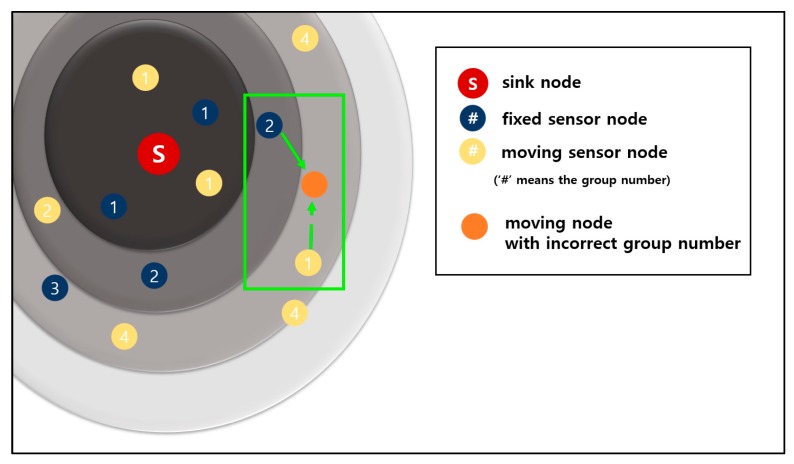
Incorrect group number setting scenario and its resolution.

Figure 15 shows an example of preventing a sensor node from setting an incorrect group number. The dark blue circles are fixed sensor nodes, and the yellow circles are mobile sensor nodes.

The orange circle in the green rectangle is a moving sensor node that wants to obtain a group number. The orange sensor node receives advertisement packets from a fixed sensor node of group number 2 and a moving sensor node of group number 1. The group number information of a moving sensor node is smaller than that of a fixed sensor node.

In the case of the original 3-D GM-MAC method, the orange sensor node sets the group number by using the information of the moving sensor node. However, this is incorrect information because the moving sensor node set the group number a long time ago and has since moved a long distance. This propagates incorrect group number information.

In contrast, the newly proposed method chooses the correct information. This method puts the information of the fixed sensor node ahead of that of the moving sensor node. In the case of Figure 15, the orange sensor node chooses the information of the fixed sensor node. The fixed sensor node does not move, and this information is accurate.

### 3.3. Advanced Group Number Management

The original 3-D GM-MAC uses a weight for the moving distance and group number when resetting. However, when a group number is set up by using the calculated value of this equation, the group number is incorrectly set in some cases.

**Figure 16:**
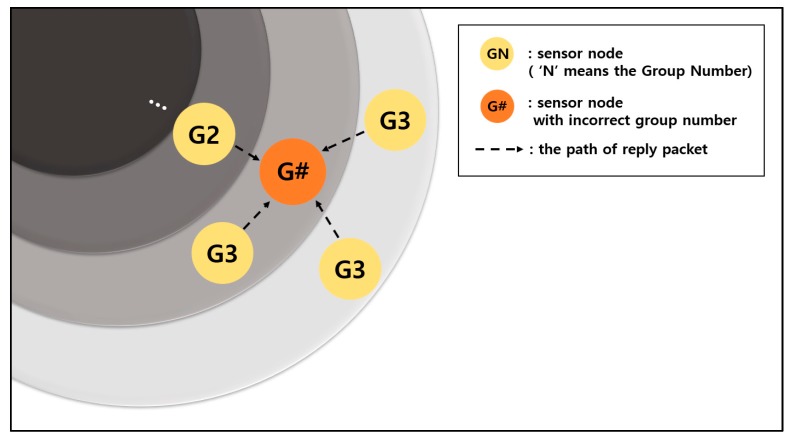
Incorrect group number resetting scenario.

Figure 16 shows an example whereby an incorrect group number is set up when the group number is reset using the original method. The orange node is a node that wants to reset the group number.

This node sends a Hello packet to other sensor nodes within its communication range. The sensor nodes that have received the Hello packet send Reply packets containing their group number information. The dotted lines in the figure are the transmission paths of the Reply packets.

In Figure 16, the orange node receives the information for group number 2 once and the information for group number 3 three times. If the equation introduced in Section 2.2. of this paper is used, the group number is set as 4. However, group number 2 does not exist in the communication range. As a result, the sensor nodes with the group number 3, including the orange node, are placed in a situation where data transmission is impossible.

If communication cannot be performed because of an incorrect group number is set, new data continuously accumulate in the buffer. Furthermore, a delay occurs in data collection. From the aspect of energy, the corresponding node wastes energy because it keeps attempting and failing to transmit data. In addition to attempts made to transmit data with incorrect information, energy can be wasted to resend Hello packets and receive Reply packets. If a CTS packet is not received for three RTS packets, the process of sending the Hello packet must be restarted.

This problem can be solved by the method proposed here. To obtain a new group number, a sensor node sends a Hello packet and receives Reply packets from surrounding nodes. Then, it sets its group number by adding 1 to the smallest number among the group numbers of Reply packets. The smallest group number is assigned regardless of the weights of moving distance, maximum movable distance, and group number.

In addition, entire group numbers are reset periodically to provide reliability. Resetting of the entire group numbers means that every group number is reset by using the same method as the initial group number resetting for all sensor nodes. If the smallest group number is assigned unconditionally without the entire group number resetting, when a group number is set incorrectly, that error propagates and affects the entire system. Resetting of the entire group numbers prevents such a problem.

**Figure 17:**
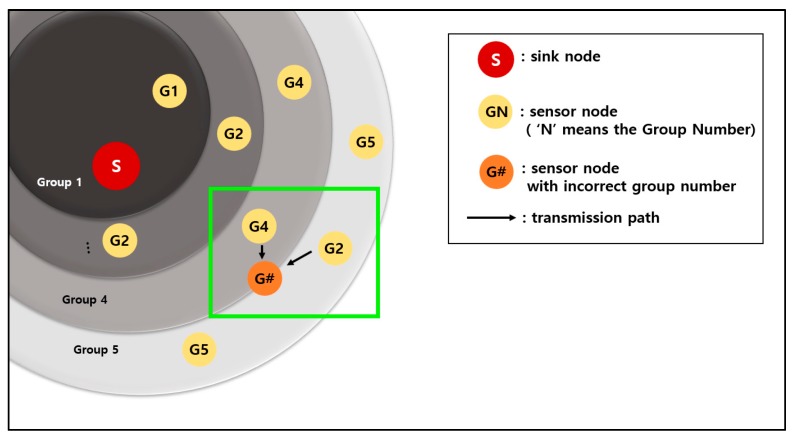
Group number resetting scenario.

Figure 17 shows an example that requires the resetting of entire group numbers. In the figure, the orange node in the green box is a node that wants to reset the group number. If the method proposed is applied, the orange node chooses the information of number 2, which is the smallest value among the numbers of the Reply packets. As the smallest group number is 2, the orange node’s group number is set as 3. However, in this case, because the node number 2 that sent the information has moved a lot from its original area, the information is incorrect. Currently, the G2 node has deviated greatly from the area of its group 2. As there is no node with the group number 1 in its vicinity, data transmission is impossible.

In a situation like the above example, resetting of the group numbers for entire nodes must be performed periodically to provide reliability. Group number resetting for entire nodes means that the group numbers that are already set up are all deleted, and a procedure similar to that of the initial group number setting is performed. This was introduced in Section 2.1.

The proposed method simplifies the procedure of the group number resetting. Furthermore, it increases the energy efficiency by reducing the possibility of incorrect group number setting.

## 4. Simulation and Consideration

### 4.1. Simulation for Buffer Threshold

#### 4.1.1. Simulation Environment of Buffer Threshold

Table 2 shows the simulation environment of GM-MAC. It is two-dimensional, and the sensor nodes do not have mobility.

#### 4.1.2. Consideration for Buffer Threshold

Table 3 shows the comparison results between the original buffer threshold and Equation (7) in Section 3.1. Data delay was not included in the comparison factors because 3-D GM-MAC targets no data loss. In the case of an application in which it is necessary to transmit data quickly in real time, the buffer threshold can be reduced so that the data can be relayed quickly. However, in this case, it must consume a lot of energy.

In the original buffer thresholds, the first node uses up all of its energy on the 2789th day. In the proposed method, it was the 2790th day. In the energy efficiency aspect, the two equations showed almost no difference.

The strength of the new buffer threshold proposed is displayed in the data loss rate and data collection rate. In the experiment of the original buffer thresholds, the data loss rate was high, approximately 9.4143%. In contrast, there was no data loss in the newly proposed buffer threshold method. Furthermore, the data collection rate was approximately 90.5851% for the original buffer thresholds, showing that a large amount of data was not collected. However, the newly proposed buffer threshold method had a 99.9991% data collection rate, indicating that almost all of the data were collected. The portion slightly below 100% was not the lost data but the data in the process of being delivered to the sink.

**Figure 18:**
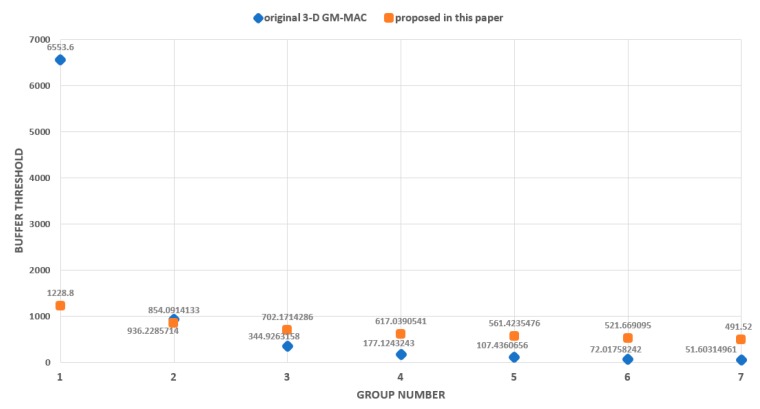
Buffer threshold.

The occurrence of large data loss in the original buffer thresholds can be explained through Figure 18. Figure 18 shows the values of the buffer thresholds according to the group number. The blue diamonds indicate the original buffer thresholds, and the orange squares indicate the newly proposed buffer thresholds. The original buffer threshold decreases rapidly as the group number increases. If the buffer threshold decreases rapidly, the buffer usage rate also decreases rapidly, and, as a result, data loss can occur easily.

### 4.2. Simulation for Using Fixed Sensor Nodes

#### 4.2.1. Simulation Environment of Using Fixed Sensor Nodes

Table 4 shows the simulation environment of 3-D GM-MAC. It is three-dimensional, and the sensor nodes have mobility.

#### 4.2.2. Consideration for Using Fixed Sensor Nodes

To evaluate the method of using fixed nodes, two experiments were conducted. In the first experiment, the number of times the group number was reset by the sensor node was compared between the original 3-D GM-MAC and the proposed method. The second experiment compared the energy consumption for all sensor nodes.

**Figure 19:**
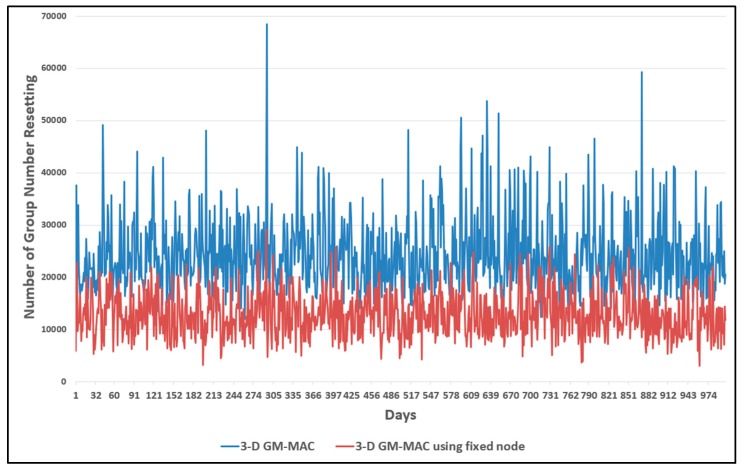
Comparison of node group number resets.

Figure 19 shows a graph for the number of times the group number was reset. The horizontal axis of the graph shows the number of days, and the vertical axis shows the number of times the group number was reset. The blue line is the result of the original 3-D GM-MAC, and the red line is the result of 3-D GM-MAC using fixed nodes, which was proposed in this paper.

When a sensor node resets the group number, it means that there is no next upper-level node within the range that the sensor node can communicate. The next upper-level sensor node means that its group number is smaller by 1. When there is no upper-level sensor node within a communication range, it means that the corresponding sensor node has an incorrect group number. This means that the group number information of the corresponding sensor node is not suitable for the located place.

The graph clearly shows that the red line resetting the group numbers was less than the blue line. Compared with the original 3-D GM-MAC, the proposed method showed that the number of times it took to reset the group number decreased by approximately 43.4%. 

**Figure 20:**
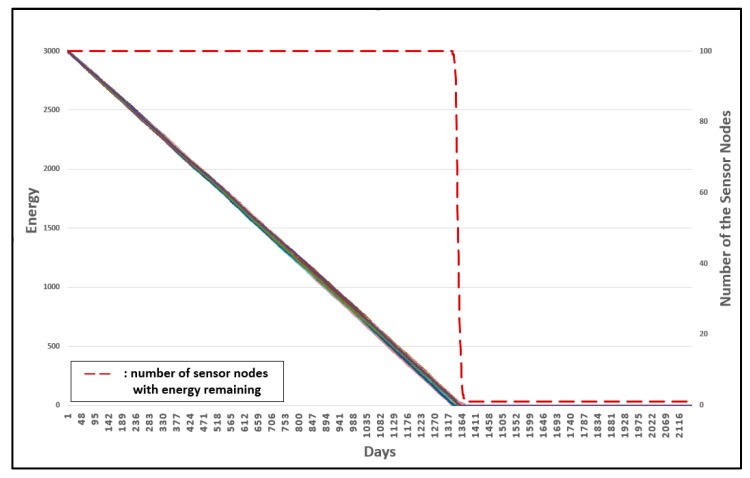
Energy consumption of 3-D GM-MAC without fixed sensor nodes.

**Figure 21:**
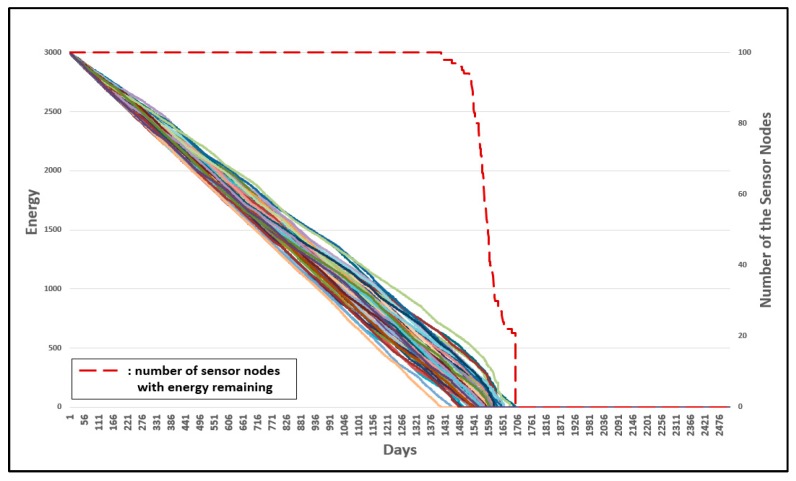
Energy consumption of 3-D GM-MAC using fixed sensor nodes.

Energy is used when resetting the group number. Therefore, as the frequency of group number resetting decreases, the energy consumption decreases. Figure 20 and Figure 21 show the results of the energy consumption decrease.

Figure 20 shows the energy consumption of all sensor nodes for the original 3-D GM-MAC. Figure 21 shows the energy consumption of all sensor nodes for the 3-D GM-MAC using fixed nodes. The left vertical axis of the graph shows the energy. The right vertical axis of the graph is the number of sensor nodes. The horizontal axis of the graph indicates the number of days. Each solid line indicates the remaining energy of the sensor nodes. The initial energy of the sensor node is 3000 mW. The red dotted line indicates the number of sensor nodes with energy remaining.

The time point at which the first node consumes all energy is approximately the 1330th day in Figure 20, whereas it is approximately the 1415th day in Figure 21. A sensor node uses energy to reset the group number, and the 3-D GM-MAC using fixed nodes showed a smaller frequency of group number resetting than the original 3-D GM-MAC. This is the reason why the 3-D GM-MAC using fixed nodes consumes less energy than the original 3-D GM-MAC. The life cycle of the sensor node increased by approximately 10%.

In Figure 20, every sensor node consumed all the energy almost simultaneously. However, in Figure 21, the sensor nodes showed different tendencies from each other in terms of energy consumption. When just one sensor node, among all of the sensor nodes, uses up all of the energy and remains in an inoperable state, it affects the entire system. It is crucial that every sensor node consumes energy with the same tendency. In the 3-D GM-MAC using fixed nodes, the sensor nodes do not show the same tendency for energy consumption, but, because the time point when the first node consumes all energy increased by about 10%, it is a significant result.

### 4.3. Simulation for Advanced Group Number Management

#### 4.3.1. Simulation Environment of Advanced Group Number Management

The simulation environment for the advanced management of group numbers was the same as that shown in Table 4. The experiment was conducted in a 3-D environment. 

#### 4.3.2. Consideration for Advanced Group Number Management

**Figure 22:**
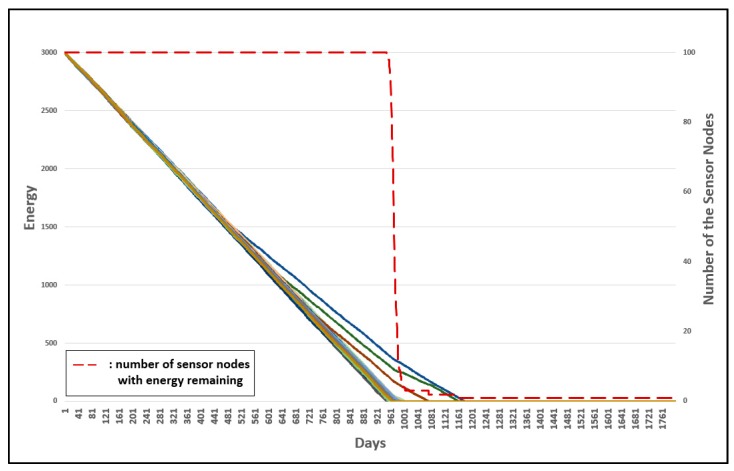
Energy consumption of original 3-D GM-MAC.

**Figure 23:**
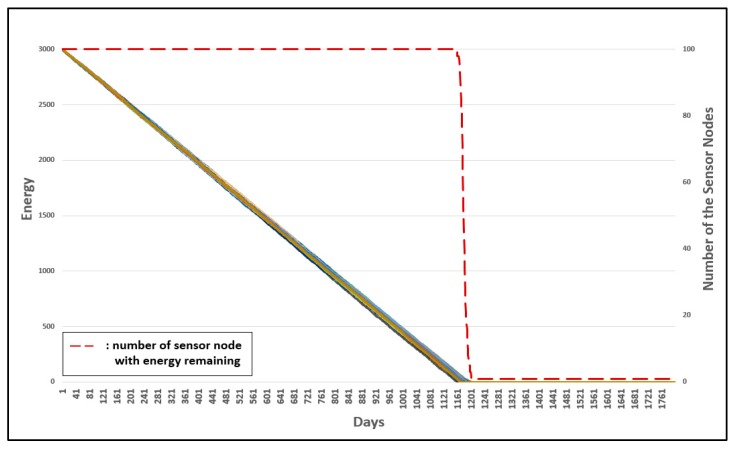
Energy consumption of 3-D GM-MAC using advanced group number management.

**Figure 24:**
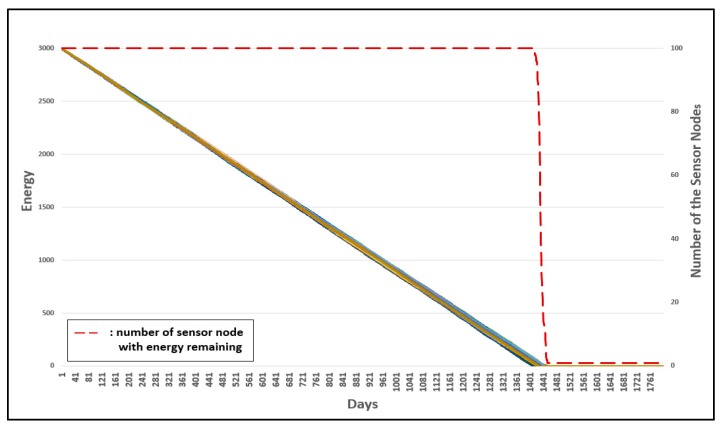
Energy consumption of 3-D GM-MAC using advanced group number management and entire group number resetting.

Three experiments were conducted to evaluate the advanced management of group numbers. Figure 22 shows the energy consumption of the original 3-D GM-MAC. Figure 23 shows the advanced management of group numbers, and Figure 24 shows the energy consumption when the advanced management of group numbers is used and all group numbers are reset periodically.

The horizontal axis shows the time whereby the sensor nodes are alive. The left vertical axis of the graph shows the energy of the sensor nodes, and the right horizontal axis shows the number of sensor nodes with energy remaining. The dotted line shows the number of sensor nodes with energy remaining. Each line indicates the energy of each sensor node.

In Figure 22, the first node to consume all the energy does so on approximately the 941th day. This occurred on about the 1161th day in Figure 23, and approximately the 1401th day in Figure 24. The result was improved by approximately 23.4% in Figure 23, when compared with Figure 22. Figure 24 shows a more improved result compared with Figure 23. Consequently, Figure 24 shows a much more improved result when compared with Figure 23. The improvement was approximately 48.9%.

If the accuracy of the group number assignment is increased, the energy efficiency increases. This is because it reduces the number of cases where the corresponding node continues to send data and fails. In addition, it is also because the energy consumption for the Hello packets and Reply packets is reduced.

As shown in Figure 24, when all group numbers are reset periodically, attempts at communication with an incorrect group number can be definitively reduced. This leads to an increase in energy efficiency.

Above all, every sensor node showed an equal energy consumption rate. When even just one sensor node used up all the energy, it could affect the entire WSN. Therefore, it is very important to have the sensor nodes consume energy at a similar rate.

## 5. Conclusions

In this paper, methods for improving the stability of 3-D GM-MAC were proposed. First, a new buffer threshold equation was derived. Several experiments were conducted to derive an equation that guaranteed energy efficiency without data loss. This is an equation for a polynomial function and a logarithmic function containing variables. As a result, Equation (7) was derived. Although the newly derived buffer threshold equation showed a similar energy efficiency to that of the original 3-D GM-MAC, the data loss rate and data collection rate were improved. The data loss rate of the newly proposed equation was 0%. The data collection rate was 99.9991%. The collection rate did not reach 100% because data were present in the middle of the path leading to the sink, not because the data were lost.

The second method of improving the stability was to use fixed sensor nodes. When the initial group numbers are set and the group numbers of entire nodes are reset periodically, the fixed sensor nodes have priority over the mobile sensor nodes with respect to the propagation of group number information. The fixed sensor nodes had very high reliability when compared with the mobile sensor nodes in terms of group number information. According to the simulation results, the number of resetting nodes decreased by approximately 43.4% when compared with the original 3-D GM-MAC. Furthermore, the energy efficiency increased by about 10%.

The final proposed method was an advanced method of group number management. For the original method of resetting group number by using an equation in which the group number and moving distance are weighted, the probability of assigning an incorrect group number is high. Instead of the original method, a method of setting the group number by adding 1 to the smallest group number information among the Reply packets was proposed. Furthermore, to provide reliability to this method, a method of periodically resetting the group numbers for all sensor nodes was proposed. In the simulation results, when the new group number resetting method was adopted, the energy efficiency increased by approximately 23.4% when compared with the original method. In addition, the adoption of periodical group number settings showed a 48.9% improvement in the end. Future work is planned to apply the buffer threshold equation to a 3-D mobility environment. 

## Figures and Tables

**Table 1:** Simulation details for deriving buffer threshold equation.

Components	Descriptions
IoT system environment (physical space)	800 m * 800 m
Number of sensor devices	1 sink, 100 sensor devices
Battery capacity	3000 mW
Tx energy consumption	0.0145 mW
Rx energy consumption	0.0156 mW
Maximum buffer size of sensor device	8192 Bytes
Maximum distance that the sensor device can communicate	90 m
Data generation	average 1 time/1 min (a Poisson Process distribution)
Number of tests	100

**Table 2:** Simulation environment of group management medium access control (GM-MAC) without mobility.

Components	Descriptions
IoT system environment (physical space)	800 m * 800 m
Number of sensor devices	1 sink, 100 sensor devices
Battery capacity	3000 mW
Tx energy consumption	0.0145 mW
Rx energy consumption	0.0156 mW
Maximum buffer size of sensor device	8192 Byte
Maximum distance that the sensor device can communicate	90 m
Data generation	average 1 time/1 min (a Poisson Process distribution)

**Table 3:** Comparison of reconfigurable buffer thresholds.

	Buffer Threshold of Original 3-D GM-MAC	Buffer Threshold of Proposed in This Paper
Time that one of the sensor nodes takes to exhaust all energy [day]	2789	2790
data loss rate [%]	9.4143	0
data collection rate [%]	90.5851	99.9991

**Table 4:** Simulation environment of 3-D GM-MAC.

Components	Descriptions
IoT system environment (physical space)	300 m * 300 m * 300 m
Number of sensor devices	1 sink, 100 sensor devices
Battery capacity	3000 mW
Tx energy consumption	0.0145 mW
Rx energy consumption	0.0156 mW
Maximum buffer size of sensor device	8192 Byte
Maximum distance that the sensor device can communicate	90 m
Data generation	average 1 time/1 min (a Poisson Process distribution)
Maximum movement speed of sensor device	5 m/min.
Movement pattern of sink and sensor device	Randomly movement
